# Interaction of Maternal Race/Ethnicity, Insurance, and Education Level on Pregnancy Outcomes: A Retrospective Analysis of the United States Vital Statistics Records

**DOI:** 10.7759/cureus.24235

**Published:** 2022-04-18

**Authors:** Oluwasegun A Akinyemi, Stella Adetokunbo, Kindha Elleissy Nasef, Olufemi Ayeni, Bolarinwa Akinwumi, Mary O Fakorede

**Affiliations:** 1 Surgery, Howard University, Washington, USA; 2 Community Medicine, Federal Teaching Hospital, Ido-Ekiti, NGA; 3 Obstetrics and Gynecology, Howard University College of Medicine, Washington, USA; 4 Health Sciences and Social Work, Western Illinois University, Macomb, USA; 5 Family Medicine, Howard University College of Medicine, Washington, USA

**Keywords:** association, pregnancy outcome, race/ethnicity, insurance, maternal education

## Abstract

Objective: The objective is to determine the association between maternal race/ethnicity, insurance, education level, and pregnancy outcomes.

Methods: We queried the U.S. vital statistics records from 2015 to 2019 to analyze all deliveries. Using a multivariate analysis model, we determined the interaction between maternal race, insurance, education, and pregnancy outcomes. The outcome measures were the 5-min Apgar score, neonatal unit admission, neonates receiving assisted ventilation > 6 hours, mothers requiring blood transfusion, and the intensive care unit admission.

Result: There were 13,213,732 deliveries that met our inclusion criteria. In the study population, 52.7% were white, 14.1% blacks, 22.9% Hispanics, and 10.4% belonged to other races. 37.5% of the women had a high school education, 49.1% had a college education, and 12.3% had advanced degrees. Black mothers with high school education were more likely to require blood transfusion following delivery than Whites at the same education level, OR=1.08 (95% CI 1.05-1.11, p < 0.05). They were also more likely to be admitted into intensive care. The difference only disappeared among blacks with advanced education (OR=1.0; 95% CI 0.89-1.12, p > 0.05). Across all races/ethnicities, private insurance and advanced education were associated with better pregnancy outcomes.

Conclusion: In the U.S., women with high socioeconomic status have better pregnancy outcomes across all races/ethnicities.

## Introduction

While the United States spends more on childbirth than any other country globally, its pregnancy outcomes are worse than in other high-resource countries and even worse for Black and Native American women. The most recent U.S. maternal mortality ratio of 20.1 per 100,000 pregnancies represented approximately 754 maternal deaths in 2019, with this ratio varying widely across different states [1]. Comparing this CDC figure to other countries in the World Health Organization's latest maternal mortality ranking, the U.S. would rank 55th, just behind Russia (17 per 100,000) and just ahead of Ukraine (19 per 100,000) [2]. Likewise, the U.S. pregnancy outcomes are worse than in other developed countries [3]. Its infant mortality rate is 5.79 deaths per 1,000 live births, with significant racial disparities [4]. Studies show that socioeconomic disadvantage is linked to a higher risk of adverse birth outcomes in the U.S. and other highly industrialized countries [4].

Social determinants of health (SDH) such as maternal education and access to insurance significantly impact pregnancy outcomes. Studies have shown that low maternal education is associated with poor pregnancy outcomes [5]. Access to health insurance, especially during pregnancy, can affect the health of both the mother and the child. In many U.S. states, low-income women do not qualify for Medicaid until pregnant [6]. Medicaid has long played an essential role in providing maternity-related services for pregnant women, paying for nearly half of all births in the United States (NGA 2014) [6]. Uninsured women are more likely to have poor outcomes during pregnancy and delivery than are women with insurance [7]. Uninsured new mothers would benefit from consistent access to coverage and care expanding Medicaid would provide [7]. This is particularly relevant given the COVID-19 pandemic and the ensuing economic crisis, putting even more women at risk of losing their insurance before, during, and after pregnancy [8]. Despite these reports, studies have not demonstrated an improvement in maternal outcomes related to health insurance alone, and evidence of improvements in outcomes for newborns as a result of increased population health insurance rates is inconclusive [7]. The present study explored the interaction between maternal race, educational status, and insurance type and how these interactions affect pregnancy outcomes across five measures.

The article was presented at the Annual Academic Meeting 2022 Joint RCOG/Blair Bell Research Society Meeting of the Royal College of Surgeons in February 2022.

## Materials and methods

Study design and data sources

We extracted the data for this study from the US Vital Statistics Records 2015-2019. The National Vital Statistics Records database provides complete information regarding births and deaths in the United States. In addition, it contains annual reports that present detailed vital statistics data, including natality, mortality, marriage, and divorce [9].

We explore the association between SDH and pregnancy outcomes by breaking down SDH into maternal race/ethnicity, insurance type, maternal education level, and pregnancy outcomes into five measures. These measures are 5-min Apgar score, neonatal unit admission, neonates receiving assisted ventilation for longer than six hours, mothers requiring blood transfusions, and the intensive care unit admission. Since the database is de-identified and publicly available, ethical clearance or Institutional Review Board approval was not necessary.

Study population

Among the 13,213,732 deliveries that fulfilled our study criteria, 52.7% were Caucasian, 14.1% were Black, 22.9% were Hispanic, and 10.4% belonged to other races/ethnicities. In addition, among the studied population, 37.5% had a high school education, 49.1% had a college education, and 12.3% had advanced degrees.

Patient characteristics and risk factors

In this study, we defined maternal education level as either high school, college, or advanced education. In addition, race/ethnicity was stratified as White, Black, Hispanic, and other, while the Insurance status was divided into uninsured, public insurance, and private insurance.

Definition of study outcome

They were five primary measures of pregnancy outcome. These are the 5-min Apgar score ( defined as Apgar score < 7), neonatal unit admission, neonates receiving assisted ventilation for ≥ 6 hours after delivery, mothers requiring blood transfusion, and maternal admission to the intensive care unit.

Statistical analysis

We expressed categorical variables as frequencies and percentages and continuous variables as mean ± standard deviations. Chi-square and the independent sample t-test were utilized to compare categorical and continuous variables.

Utilizing a multivariate analysis model, we determined the interaction between maternal races/ethnicities, maternal insurance type, and maternal education and how these interactions affect pregnancy outcomes across the five measured outcomes. We control for covariates such as maternal age, pre-pregnancy diabetes, hypertension, pre-pregnancy obesity, Cesarean section (CS), previous cesarean section ( PCS), augmentation and labor induction, and delivery weight. A two-tailed p-value < 0.05 was regarded as statistically significant. All statistical analyses were performed using the STATA 16 (StataCorp College Station, TX).

## Results

Table 1 shows the baseline distribution of the pregnancy outcome measures. The neonatal admission rate was 8.86% in the study population.

**Table 1:** Baseline distribution of outcome measures

	Frequency	Percentages
Neonatal Unit Admission (NICU)	1,170,392	8.86
5-min Apgar Score (Apgar)	262,407	1.99
Assisted Ventilation > 6hours (AsVent)	194,218	1.47
Maternal transfusion (MatTransf)	50,387	0.38
Intensive care Admission (InCare)	20,924	0.16

Table 2 shows the distribution of the maternal education level stratified by race/ethnicity. In about 77.89% of the Hispanic population, the highest education level was High school education. Among women with an advanced level of education, 15.91% were whites, 5.67% were Blacks, and 4.18% identified as Hispanics.

**Table 2:** Baseline distribution of maternal education level by race/ethnicity

Variables	White (N=6,958,926)	Black (N=1,859,931)	Hispanic (N=3,021,908)	Others (N=1,366,873)	P-value
High School	47.63	75.19	77.89	41.58	<0.001
College	36.01	18.45	16.84	32.52	<0.001
Advanced	15.91	5.67	4.18	20.76	<0.001
Unknown	0.46	0.69	1.10	5.14	<0.001

Table 3 shows the baseline distribution of maternal insurance type by race/ethnicity. Among the public insurance group, black women have the highest rate at 66.05%, while only 29.66% of the white population utilizes public insurance and 58.87% of Hispanics. The majority of the white women (63.37%) utilize private insurance, while only 27.5% of blacks and 29.24% of Hispanics have private insurance. Hispanic women have the highest uninsured rate of 7.00%.

**Table 3:** Baseline distribution of maternal insurance by race/ethnicity

Variables	White (N=6,958,926)	Black (N=1,859,931)	Hispanic (N=3,021,908)	Others (N=1,366,873)	P-value
Public	29.66	66.05	58.87	33.35	<0.001
Private	63.37	27.52	29.24	56.71	<0.001
Uninsured	2.93	2.74	7.00	4.93	<0.001
Others	4.04	3.69	4.90	5.01	<0.001

Table 4 shows the interaction between race and education level and how this interaction affects pregnancy outcomes across the different outcomes measures. Again, with increasing education level, there appears to be an improvement in pregnancy outcomes, especially among Hispanics, Black, and other races.

**Table 4:** Interaction between maternal race/ethnicity, education level, and pregnancy outcome † Model adjusted for age, pre-pregnancy diabetes, hypertension, pre-pregnancy obesity, CS, PCS, Augmentation, induction of labor, delivery weight, *p < 0.05

Variables	NICU	AsVent	Apgar	MatTransf	InCare
White x High School	Reference				
White x College	0.93 (0.93-0.94)*	0.96 (0.95-0.98)*	0.89 (0.88-0.90)*	0.90 (0.87-0.92)*	0.88 (0.85-0.92)*
White x Advanced	0.93 (0.92-0.94)*	0.90 (0.88-0.92)*	0.79 (0.78-0.81)*	0.98 (0.94-1.01)	0.89 (0.84-0.93)*
Black x High School	0.97 (0.97-0.98)*	0.72 (0.71-0.73)*	1.15 (1.14-1.16)*	1.08 (1.05-1.11)*	1.09 (1.05-1.14)*
Black x College	0.95 (0.94-0.96)*	0.68 (0.66-0.70)*	1.08 (1.05-1.10)*	0.90 (0.85-0.95)*	1.08 (1.01-1.15)*
Black x Advanced	0.92 (0.90-0.94)*	0.67 (0.64-0.70)*	1.04 (1.00-1.08)*	0.93 (0.85-1.01)	1.00 (0.89-1.12)*
Hispanic x High School	0.95 (0.94-0.96)*	0.65 (0.64-0.66)*	0.65 (0.64-0.66)*	0.99 (0.97-1.02)	1.00 (0.96-1.03)
Hispanic x College	0.93 (0.92-0.94)*	0.64 (0.62-0.65)*	0.62 (0.60-0.63)*	0.91 (0.87-0.95)	1.08 (1.02-1.15)
Hispanic x Advanced	0.91 (0.89-0.93)*	0.63 (0.59-0.66)*	0.58 (0.55-0.61)*	0.91 (0.83-0.99)	1.07 (0.96-1.20)
Others x High School	0.97 (0.96-0.98)*	0.81 (0.79-0.83)*	0.91 (0.89-0.93)*	1.21 (1.16-1.26)*	1.25 (1.19-1.32)*
Others x College	0.82 (0.81-0.83)*	0.57 (0.55-0.59)*	0.67 (0.65-0.69)*	0.87 (0.83-0.92)*	1.03 (0.96-1.10)
Others x Advanced	0.79 (0.78-0.80)*	0.54 (0.52-0.57)*	0.61 (0.59-0.63)*	0.87 (0.82-0.93)*	0.99 (0.91-1.07)

Table 5 shows the interaction between race and insurance type and how this interaction affects pregnancy outcomes. Private insurance has the best outcome across all races/ethnicities. Among Blacks, mothers with public insurance have better outcomes than those without insurance. Conversely, women without insurance have the worst pregnancy outcomes across all races/ethnicities.

**Table 5:** Interaction between maternal race/ethnicity, insurance type, and pregnancy outcomes † Model adjusted for age, pre-pregnancy diabetes, hypertension, pre-pregnancy obesity, CS, PCS, Augmentation, induction of labor, delivery weight, *p < 0.05

Variables	NICU	AsVent	Apgar	MatTransf	InCare
White x Public	Reference				
White x Private	0.93 (0.93-0.94)*	1.07 (1.05-1.08)*	0.94 (0.93-0.96)*	0.87 (0.85-0.90)*	0.77 (0.74-0.80)*
White x Uninsured	0.79 (0.77-0.81)*	1.07 (1.02-1.11)*	1.19 (1.15-1.23)*	1.21 (1.13-1.30)*	1.21 (1.09-1.33)*
Black x Public	0.96 (0.96-0.97)*	0.73 (0.71-0.74)*	1.16 (1.14-1.17)*	1.08 (1.05-1.12)*	1.08 (1.03-1.13)*
Black x Private	0.93 (0.92-0.94)*	0.76 (0.74-0.78)*	1.13 (1.11-1.15)*	0.92 (0.88-0.96)*	0.94 (0.88-1.00)
Black x Uninsured	1.11 (1.07-1.14)*	0.86 (0.81-0.92)*	1.43 (1.37-1.51)*	1.10 (0.97-1.24)*	1.27 (1.09-1.48)*
Hispanic x Public	0.95 (0.95-0.96)*	0.65 (0.64-0.67)*	0.65 (0.64-0.66)*	1.06 (1.02-1.09)*	1.05 (1.00-1.09)*
Hispanic x Private	0.88 (0.88-0.89)*	0.69 (0.68-0.71)*	0.65 (0.64-0.67)*	0.81 (0.78-0.84)*	0.87 (0.83-0.92)*
Hispanic x Uninsured	0.81 (0.80-0.83)*	0.73 (0.69-0.76)*	0.74 (0.71-0.77)*	0.96 (0.89-1.02)	0.88 (0.79-0.97)*
Others x Public	1.01 (0.99-1.02)	0.82 (0.79-0.84)*	0.92 (0.90-0.94)*	1.18 (1.13-1.23)*	1.19 (1.12-1.26)*
Others x Private	0.82 (0.81-0.83)*	0.67 (0.66-0.69)*	0.75 (0.73-0.77)*	0.85 (0.82-0.89)*	0.91 (0.86-0.96)*
Others x Uninsured	0.54 (0.52-0.56)*	0.54 (0.49-0.59)*	0.70 (0.65-0.75)*	0.92 (0.82-1.04)	0.94 (0.80-1.11)

## Discussion

The United States (U.S.) has continued to experience disparity in the outcomes of pregnancies across many demographic strata. These disparities in pregnancy outcomes may be attributable to SDHs such as access to insurance, quality of education, and race [10]. The present study explored the association between race/ethnicities, insurance type, maternal education level, and pregnancy outcome measured across five parameters.

NICU admissions

Increase NICU Admission Among White Mothers

In the present study, infants of white mothers have an elevated rate of NICU admission. This finding is consistent with the result obtained by Horbar et al., which explored 743 NICUs across the U.S and reported that infants of white mothers accounted for the highest NICU admission rates of 46% (53,895) [11]. Sigurdson et al. reported that black women have worse neonatal outcomes with higher NICU admission rates than white women [12]. This difference in outcomes was because Sigurdson et al. utilized a high black-serving facility with lower quality health care delivery, poorer working environments, and understaffed nursing care.

Babies Whose Mothers’ Highest Education Level Is High School Education Have the Worst Outcome

Maternal education is an essential measure of socioeconomic status (SES), with studies demonstrating improved pregnancy outcomes with increasing SES [13]. In addition, a high level of maternal education is associated with increased acquisition of health information and the ability to utilize such knowledge and access to improved health care [14]. Our study revealed that babies whose mother's highest education level is High school education have the worst outcome and are more likely to be admitted to NICU. This implies that the lower the maternal educational status, the poorer the outcome with a higher risk of NICU admission. This finding aligns with results obtained by Dingemann et al., which reported that low maternal educational level is associated with adverse outcomes in neonates, including NICU admission. Cantarutti et al. also reported the association between maternal education and pregnancy outcome [15]. In 2017, they studied 383,103 live births and reported that low maternal education and maternal birthplace are crucial contributors to adverse neonatal outcomes in Italy [15].

Babies Whose Mothers Have Public Insurance Have the Highest Risk of Neonatal Admission

In the U.S, insurance status (public, private, and uninsured) is an essential defining factor of health care access and survival [16]. Women with private insurance are associated with improved neonatal outcomes compared to uninsured women or public insurance [16]. Our study highlighted this association as women with public insurance have a higher risk of NICU admission. Private insurance is a protective factor against NICU admission among infants of non-Hispanic Whites and Hispanic mothers compared to their non-Hispanic Black counterparts. In the same vein, Greiner et al. reported that mothers on public insurance had more neonates requiring NICU admission compared to those on private insurance [17]. This was explained by a higher prevalence of pregnancy-related complications such as eclampsia and severe hypertension in the public insurance group. However, Einarsdóttir et al. declared that despite having delayed unassisted breathing establishment and a higher probability of being assigned lower Apgar scores, mothers on public insurance have a lower likelihood of being admitted to NICU than those on private insurance [18]. Unlike our study, they focused only on preterm births (32-36 weeks). These differences in sample size and demographic characteristics of the mothers and neonates may be responsible for the contrary results.

Assisted ventilation

White Mothers Are More Likely to Have Babies That Will Require Assisted Ventilation for More Than Six Hours Following Delivery

Respiratory failure in neonates is a critical and relatively common problem many newborns encounter requiring ventilation assistance [19]. Assisted ventilation in neonates is a life-saving procedure for regular gaseous exchange and lung function. We found that babies of white mothers had the highest odds of requiring assisted ventilation ≥ 6 hours following delivery. This is consistent with the findings of Ondusko et al. In a retrospective study aimed at determining racial disparities in Antenatal corticosteroids (ACS) use and outcomes; they reported that neonates of black women had a significantly lower probability of receiving ACS, surfactant therapy, as well as assisted ventilation ≥ 6 hours after delivery compared to those of white women [20]. Likewise, Tanner et al. reported that black mothers (in preterm births) have a lower risk of adverse neonatal outcomes such as low Apgar scores, neonatal seizures, and assisted ventilation ≥ 6 hours compared to their white counterparts [21]. However, when considering the outcomes for all neonates: preterm and term babies, Tanner et al. reported a higher number of adverse neonatal outcomes, including assisted ventilation ≥ 6 hours in black mothers than their white counterparts [21]. This finding may be due to Tanner et al.'s evaluation of mothers with higher educational attainment only, instead of evaluating a combination of higher and lower educational status as in our study.

Mothers Whose Highest Level of Education Is High School Education Have the Highest Risk of Having Babies That Will Require Assisted Ventilation ≥ 6 Hours After Delivery

As noted above, maternal educational attainment is an essential measure of SES, with maternal education and SES associated with improved birth outcomes [13]. Dingemann et al. reported that low maternal education is associated with an increased risk of adverse neonatal outcomes, including NICU admission and perioperative mechanical ventilation. This finding is similar to our result and reiterated the association between maternal education level and assisted ventilation following delivery.

Low 5-min Apgar score

Increasing Maternal Education Level Is Associated With Better Apgar Scores

Our study investigated the potential association between the 5-min Apgar scores and maternal education. Our results revealed that mothers whose highest level of education was high school have the highest risk of babies with low 5-min Apgar scores. This result aligns with Almeida et al. They studied about 12 million live births and reported that the risk of a low 5-min Apgar score decreases markedly as maternal education level increases [22]. This implies that mothers with lower educational status have a higher probability of delivering babies with low 5-min Apgar scores. In another study, Odd et al. reported that increasing maternal education level is associated with a significant reduction in the odds of low 1 or 5-min Apgar scores in their newborns [23].

Black Mothers Have the Highest Likelihood of Having Newborns With a Low 5-Min Apgar Score

In the present study, babies of black mothers were more likely to have a low 5-min Apgar score than other races/ethnicities supporting Profit et al., who also reported the highest risk of a low 5-min Apgar to score among Black mothers [24].

Uninsured Women More Likely to Have Low Apgar Score Babies

Our analysis revealed that uninsured patients are more likely to have babies with low 5-min Apgar scores than private or public insurance women. This finding is consistent with the result of Einarsdóttir et al., who reported that mothers with public insurance were more likely to deliver babies with a low 5-min Apgar score than those on private insurance [13]. In another study, Lai et al. reported that maternal public insurance status is a significant predictor of low 5-min Apgar scores following delivery [25]. The association between lack of insurance and a lower Apgar score is not unexpected considering that access to insurance is associated with improved health outcomes in the United States.

Maternal blood transfusion

Black Mothers Are More Likely to Receive a Blood Transfusion During Pregnancy Compared to Other Races/Ethnicities

The United States continues to experience significant disparities in maternal morbidity and mortality following delivery. In the last three decades, there has been a steady rise in severe maternal morbidity (SMM) such as hemorrhage, strokes, and embolism in the country [26]. The increasing rate of these complications is more predominant in the minority population, resulting in higher maternal morbidity and mortality rates [26]. Mothers requiring blood transfusion is a proxy for an increased risk of PPH, one of the most common causes of increased maternal morbidity and mortality [26]. In our study, black mothers were more likely to require blood transfusion following delivery. This study finding is similar to that of Holliman et al., who reported higher odds of blood transfusion among black women compared to other races/ethnicities [27].

Mothers Whose Highest Level of Education Is High School Education Were More Likely to Require a Blood Transfusion During Pregnancy

Moreira and Gubert reported that mothers with lower educational status, such as those who did not have more than eight years of education, have an increased risk for SMM, including blood transfusion [28]. Our finding agrees with this finding. Furthermore, we found that SMM such as blood transfusion is commonest among mothers whose highest educational level is high school.

Mothers With Private Insurance Are Less Likely to Receive a Blood Transfusion During Pregnancy

In our study, women with public insurance or uninsured women have the highest risk of requiring blood transfusion during pregnancy. This aligns with other findings in this study associating private insurance with better health outcomes. A similar finding was reported by Conrey et al., who reported the highest risk of receiving blood transfusion among public insurance or uninsured women [29].

Admission to intensive care

Blacks and Hispanic Mothers Have the Highest Risk of Admission to Intensive Care Unit Following Delivery

The present study reinforced the increasing disparity in pregnancy outcomes in the U.S., with black women having the highest risk of admission to intensive care units. Since black women have a higher rate of requiring blood transfusion, it is not unexpected that they are more likely to be admitted into the ICU. The finding agrees with Lin et al. who reported the highest risk of ICU admission among black women [30].

Mothers With Private Insurance Are Less Likely to Be Admitted to Intensive Care Units Following Delivery

Finally, our study reported that Women with public insurance or uninsured women were more likely to be admitted to the ICU than women with private insurance. This highlights the importance of access to quality health insurance in improving pregnancy outcomes and women’s health in the United States. This finding supports earlier results in our study associating private insurance with better pregnancy outcomes among all women.

Strengths and limitations

A significant strength of this study is the large sample size which increases our ability to detect differences when they exist. Also, we utilize the United States Vital Statistics records, which is the most successful example of inter-governmental data sharing in Public Health. However, we acknowledge potential shortcomings which may include insufficient clinical specificity in medical documentation that can affect study designs.

## Conclusions

In the present study, white mothers with advanced education and access to private insurance have the best pregnancy outcomes. Among minority populations, women with advanced maternal education and access to private insurance also have the best pregnancy outcomes. These findings highlight the importance of SDH on pregnancy outcomes in the United States. The present study supports evidence in the literature suggesting that strategies to improve pregnancy outcomes and women’s health in the United States should explore ways of reducing these disparities.
